# Application of Pathfinding Algorithms in Partial Discharge Localization in Power Transformers

**DOI:** 10.3390/s24020685

**Published:** 2024-01-21

**Authors:** Chandra Prakash Beura, Jorim Wolters, Stefan Tenbohlen

**Affiliations:** Institute of Power Transmission and High Voltage Technology (IEH), University of Stuttgart, 70569 Stuttgart, Germany; cpbeura@gmail.com (C.P.B.); jorimwolters@gmail.com (J.W.)

**Keywords:** power transformer, partial discharge, UHF, localization, condition monitoring, pathfinding algorithm

## Abstract

The introduction of artificial intelligence (AI) to ultra-high-frequency (UHF) partial discharge (PD) monitoring systems in power transformers for the localization of PD sources can help create a robust and reliable system with high usability and precision. However, training the AI with experimental data or data from electromagnetic simulation is costly and time-consuming. Furthermore, electromagnetic simulations often calculate more data than needed, whereas, for localization, the signal time-of-flight information is the most important. A tailored pathfinding algorithm can bypass the time-consuming and computationally expensive process of simulating or collecting data from experiments and be used to create the necessary training data for an AI-based monitoring system of partial discharges in power transformers. In this contribution, Dijkstra’s algorithm is used with additional line-of-sight propagation algorithms to determine the paths of the electromagnetic waves generated by PD sources in a three-dimensional (3D) computer-aided design (CAD) model of a 300 MVA power transformer. The time-of-flight information is compared with results from experiments and electromagnetic simulations, and it is found that the algorithm maintains accuracy similar to that of the electromagnetic simulation software, with some under/overestimations in specific scenarios, while being much faster at calculations.

## 1. Introduction

Power transformers are the nodes that regulate our electric grids, without which modern society could not thrive in the way it has been doing for the past century. The importance of these transformers is not to be underestimated. Though small transformers failing is nothing but a nuisance, the prospect of failure in larger power transformers, like the ones referred to in this paper, must be actively avoided. With these transformers being the feed-in points for parts of cities and entire towns, catastrophic failure in one of them could mean the halting of modern society for the subsequent part of the grid. For this reason, monitoring of power transformers is an integral part of their design and functioning. One of the key reasons for transformer failure is partial discharge (PD) activity; hence, such activity must be detected and localized as soon as possible [1].

Various methods are available for PD monitoring, such as the conventional electrical method based on IEC 60270 [2]. However, online monitoring methods such as the ultra-high-frequency (UHF) method also have the advantage of localizing the PD sources [3,4,5]. One widely used localization method is the time difference of arrival (TDOA) of the electromagnetic signals at the UHF sensors. However, there is an inaccuracy in localization because of the complex propagation paths inside power transformers [6].

With the advent of artificial intelligence (AI), there is the possibility of improving and automating PD localization and differentiating between different PD types [7]. Localization is a type of regression problem where the TDOA information obtained from four sensors can be given as input to an artificial neural network (ANN) to predict the x, y, and z coordinates of the PD source [8,9]. Additionally, localization errors from TDOA can also be corrected by using multiple radial basis function (RBF) neural networks [10]. Determining the type of PD, however, is a classification problem, and ANNs can be used to determine the type of PD from data such as phase-resolved PD (PRPD) patterns [11,12].

However, such failure detection and prevention methods rely on significant amounts of data to be trained [13]. These training data are difficult to come by, requiring vast amounts of time to be collected experimentally or otherwise requiring the setup of computationally expensive and laborious simulations to create such data [14]. In any case, gathering the data needed for these machine learning models to succeed is a strenuous endeavor, often accompanied by inaccuracies and variations depending on the testing platform. 

Pathfinding algorithms can also obtain the TDOA information required to localize PD sources using ANNs. Such algorithms find the shortest route between two points and are generally used in games for AI [15]. However, they can also be used to find the shortest path between the PD source and the UHF sensor in a transformer [16,17].

This paper explores the possibility of utilizing pathfinding algorithms to quickly create the aforementioned data while being accurate enough for artificial intelligence to use the data created. Research has been conducted on applying a time difference of arrival (TDOA) database for localization [17]. First, a pathfinding algorithm is used on a three-dimensional simulation model to calculate the TDOA of the signals from PD sources to UHF sensors. Next, the TDOA information and the PD coordinates are stored in a database. Subsequently, the TDOA database is used as a lookup table for TDOAs measured during operation, thus predicting the coordinates of the PD sources. However, the research so far has certain limitations and scope for improvement. First, using Dijkstra’s algorithm [18] for pathfinding adds inaccuracy over longer propagation distances [19], as explained in the following sections. Additionally, the simulation and experiments are carried out in test tanks or smaller transformers. Most importantly, the database cannot be used to localize PD occurring inside the winding. In this contribution, an improved pathfinding algorithm is developed and tested using a three-dimensional computer-aided design (CAD) model of a 300 MVA transformer, focusing on resolving the inability to localize PD occurring inside the winding. The accuracy of the developed algorithm is then compared to the experimental and simulation results obtained from the transformer and electromagnetic simulations, respectively.

## 2. Experimental and Simulation Setup

In a previously conducted experiment, 24 holes were drilled into a 300 MVA, 420 kV transmission transformer with tank dimensions of 900 cm × 400 cm × 260 cm. In each of these holes, wire monopole antennas with a length of 10 cm each were installed. Holes were drilled on the front, rear, and side tank walls to allow for complete coverage of the tank. The oil was drained from the tank to drill holes, and testing was carried out in an air-filled tank [20].

**Figure 1:**
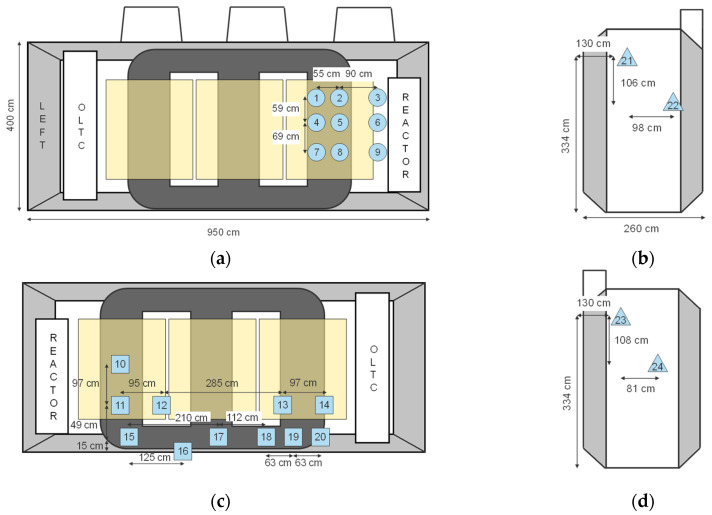
Sensor placement of all 24 sensors utilized in the experiment, the simulation, and the testing of the algorithm on the (**a**) front tank wall; (**b**) left tank wall; (**c**) rear tank wall; (**d**) right tank wall [20].

**Figure 2:**
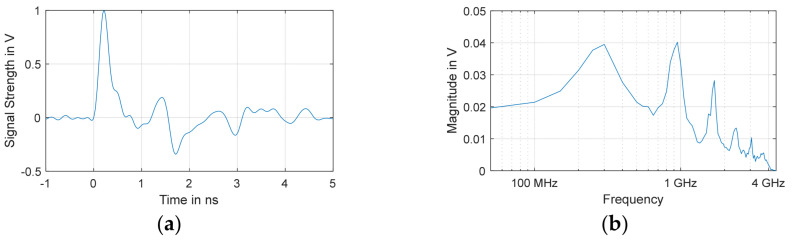
Artificial PD pulse in the (**a**) time-domain; (**b**) frequency-domain (FFT) [20].

The positions of all monopole antennas are shown in Figure 1. A total of 4 antennas were used as receiving sensors, and 21 were used as artificial PD sources, resulting in 84 measurements. In this case, following the best practices of sensor positioning and according to the results of previous research [21], sensors 6, 13, 22, and 23 were used as receivers, and the rest of the sensors were used as signal sources. An artificial pulse generator [22] was used to generate the excitation pulse, as shown in Figure 2, which was sent to the source monopole antenna. The pulse has a short rise time of approximately 80 ps and an impulse width of approximately 200 ps with a bandwidth of approximately 1 GHz. The time-domain waveform of the input pulse was recorded by connecting the output of the pulse generator to a channel of a digital storage oscilloscope (DSO), and the remaining channels of the DSO were used to record the signals from the receivers. More information about the experiment can be found in [20].

A simulation model of the transformer has been validated in previous research [20,23]. The model was built in CST Microwave Studio to study the signal propagation and attenuation characteristics. The simulation was conducted in an air-filled tank, with the input pulse shown in Figure 2, to maintain parity with the measurements. Individual discs of the HV winding were modeled so that PD occurring in each disc of the HV winding could be localized. The monopoles were positioned at the same locations as in the experiment.

## 3. Pathfinding Algorithm

The pathfinding procedure consists of multiple steps. First, the three-dimensional simulation model of the transformer is voxelized to create the voxel grid required for the pathfinding algorithms. A voxel can be considered to be the three-dimensional equivalent of a pixel. The voxelization of the input 3D models resulted in difficulty, especially given the size of the models introduced. Voxelizing a 3D environment is a complex process, and the Python libraries utilized proved either too slow or inaccurate. For this reason, a voxelizer created by Arjan Westerdiep [24], which includes a web app for testing, was chosen for utilization. This voxelizer created accurate representations of the 3D models inserted in a timely and computationally cheap manner. The variety of export options for the voxelated environment enabled the testing of various formats to create the utilized environment, of which, ultimately, a .txt file was utilized, which allowed for quick imports and exports due to its small size and unparalleled ease of use. The resulting voxelized 3D models corresponded to an accurate representation of the power transformer with 1 cm × 1 cm × 1 cm sized voxels spanning the shell of each closed object in the space.

As a result of the voxelization process, the obtained array contains closed but hollow objects voxelized to the desired degree. The shell of these bodies is marked with a different value than the rest of the array, so a Boolean array is output with “True” values for the shell and “False” values for the empty space. All occupied cells will be practically impassable for the pathfinding algorithm, corresponding to cells that the signal waves cannot traverse. Because the algorithm will try to find a path from each of the starting points (in this case, one of the four sensors) towards every ending point (in this case, every “free” voxel not marked as impassable), all paths searching for an ending voxel inside of a shell will be caught in a loop trying to find a path towards an unreachable end. In the same way, if a signal is started from within a shell trying to find a path out, the path will bounce inside the shell until it has traversed all possible elements inside the shell.

**Figure 3:**
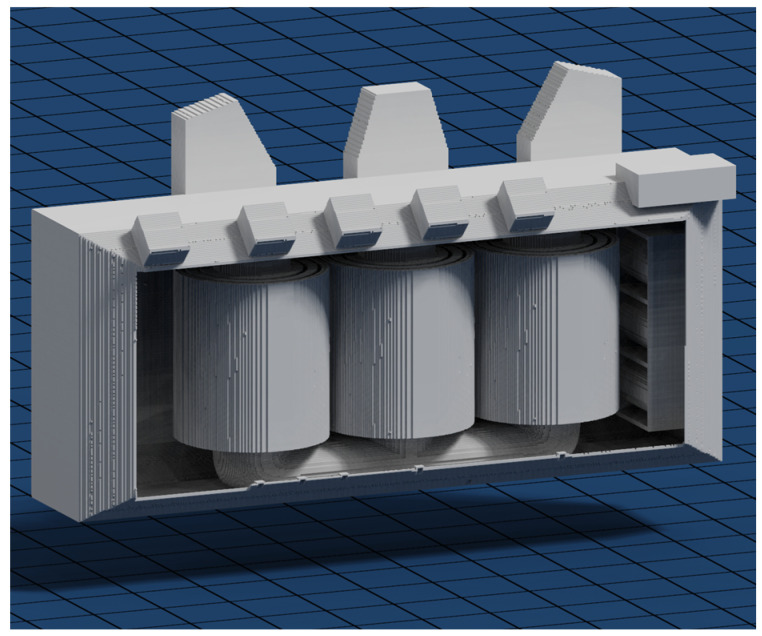
Voxelized 3D model of the transformer.

To solve this problem and account for the fact that the signal waves cannot originate inside impassable objects but rather on their surfaces, a small algorithm was designed to “fill” these empty shells with impassable voxels. This algorithm takes the same approach as the expanding wave that Dijkstra’s algorithm uses to expand the open list. This wave starts in a known empty voxel inside the power transformer’s tank but outside every other component and expands towards the neighboring voxels. Once a neighboring voxel containing a “full” value is found, the wave will not expand beyond that neighbor. This results in a mapping wave traversing every voxel inside the power transformer’s tank and bouncing off every wall it encounters. Voxels touched by this expanding wave correspond to the voxels containing oil inside the power transformer’s tank, and voxels not marked as oil cannot be traversed by PD signal waves. The wave algorithm then marks every non-oil voxel as impassable, converting the aforementioned shells into solids. The voxelized form of the 3D model is shown in Figure 3. The individual disks of the HV winding are modeled in each winding block. Hence, the time-of-flight information for PD sources inside the winding blocks can also be calculated.

**Figure 4:**
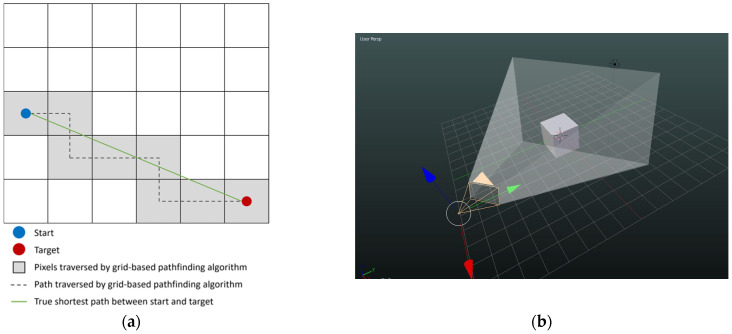
(**a**) True shortest path (in green) using Amanatides and Woo’s algorithm; (**b**) a representation of the line-of-sight check, where points obscured by the cube will not be visible from the origin [26].

In the next step, the starting point for the pathfinding algorithm is specified, i.e., the coordinates of the UHF sensor, e.g., sensor 6. Then, an algorithm developed by Amanatides and Woo is used to determine the voxels that have a direct line-of-sight to the starting point. Although not impossible, and possibly even being a more accurate approximation to the true traveled distances, the usage of curves within a voxelized environment makes the calculations and implementation in code very difficult. Using straight lines to approximate a curve is, in a basic way, very similar to the approximation made by an integration. However, using straight lines in an equidistant grid is extremely easy and fast, especially with the introduction of Amanatides and Woo’s raytracing algorithm [25], as shown in Figure 4a. With the help of this fast voxel-traversing algorithm, a new concept can be introduced: the “Line of Sight”. A line-of-sight is a simple binary check of whether a traversed voxel can “see” another traversed voxel, meaning that a straight line between both voxels is uninterrupted by obstacle voxels. This simple line-of-sight check is implemented by the Amanatides and Woo algorithm, which can quickly traverse every voxel along a straight line that connects two arbitrary points in the space without excluding small interactions between edges of the voxels and the traversed line. The optimization that this algorithm brings to the table is not to be underestimated, yet its process is simple. Based on Amanatides and Woo’s algorithm, a raytracing algorithm was coded as a simple Boolean line-of-sight check, returning True if the line-of-sight exists and False if it does not. A line-of-sight exists if the raytracing algorithm can finish traversing from start to finish without encountering a voxel that is marked as an obstacle.

In the same way, a line-of-sight does not exist if the raytracing algorithm traverses through a voxel previously marked as an obstacle. The ray-casting algorithm takes the input start and end voxels and proceeds to implement Amanatides and Woo’s algorithm but with the added condition of checking the traversed voxels for their material. If an impassable voxel is encountered along the ray, the raytracing algorithm breaks early and marks the line-of-sight between these voxels as False. Otherwise, if it finds the ending voxel, the resulting line-of-sight exists. A visual representation of the line-of-sight check is shown in Figure 4b.

Subsequently, Dijkstra’s algorithm is used for pathfinding to the voxels that do not have a direct line-of-sight to the starting point, and the resultant path is the baseline on which optimizations are made since Dijkstra’s algorithm adds an error over long propagation distances. Dijkstra’s algorithm is one of the prime algorithms used in computer science for finding the shortest path between nodes in a graph. Due to its long existence, it has frequently been used as one of the bases on which to create further algorithms. It is named after its creator, Edsger W. Dijkstra [18], a Dutch computer scientist, and was published in 1959.

The algorithm begins at a designated starting node and iteratively explores its neighboring nodes until it reaches the target node, while keeping track of the shortest distance traveled to each node. It assigns a tentative distance value to every node in the graph, which starts at infinity for all unexplored nodes. The starting node counts as an already explored node; therefore, its calculated distance to itself is zero. The algorithm then expands its calculation onto the neighboring unexplored nodes and assigns a tentative distance to each one. Its specific workings can be visualized as an expanding wave that starts at the starting node and travels away, marking every node visited and calculating the distance traveled along the graph to reach them. In reality, this happens with the help of an “open list”, which contains every node at the edge of the expansion. When the algorithm has finished assigning tentative distances to the nodes in the open list, a new expansion step is initiated. The algorithm traverses to its neighbors for every node in the expansion list, calculating the distance traveled to these new nodes and adding them to the open list. If an already expanded node is found to have a shorter path if traveled via a different path than the one already established, its tentative distance to the start and the distances of all other nodes connected to it are updated. In this way, Dijkstra’s algorithm always finds the shortest possible path to every node in the graph [27]. 

One of the strengths of Dijkstra’s algorithm is that it does not rely on a grid to perform its calculations but can also work based on a graph or a list of connected nodes. Also, a key feature is its ability to work with graphs with different weights. The connections between the nodes are called edges, which possess a weight, a cost that must be paid to travel through that connection. One notable limitation of his pathfinding algorithm is that it cannot work with negative values because it would end up causing an infinite loop. The weights of the edges, also called vertices, must always be non-negative values.

Reiterating the prime example of pathfinding algorithms, Google Maps assigns a different weight to each street based on the time it takes to traverse it and the distance traveled along that street. Closed streets are allocated a value of infinity because they cannot be traversed, while free and large streets receive small values, making them the preferred method of traveling. The time complexity of a pathfinding algorithm is measured as a function of its edges and vertices (or nodes), which makes algorithm comparisons possible and not reliant on a certain type or size of graph. Dijkstra’s algorithm works with a time complexity of O(|E| + |V|log|V|), where |E| is the number of edges and |V| is the number of vertices in the graph [28].

**Figure 5:**
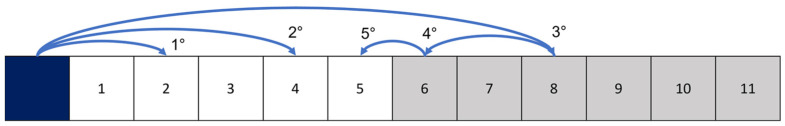
Binary jump search algorithm.

Path smoothing is performed on the baseline to increase accuracy. Path smoothing is carried out in two steps: first, a binary jump search algorithm determines the farthest voxels between which a direct line of sight exists. Now, the algorithm will start the search at voxel n + 2. If the line-of-sight check returns True, the next check will be performed at voxel n + 4. After encountering a blocked line-of-sight, the process is analogous to the previous example, where the jumps are halved until the longest straight line along the path is found. This new search algorithm always converges in 2 × *ceil*(*log*2(*l*)) *with l* ∈ ℕ steps or, in the case that the exception is found where all line-of-sight checks fail, in 2 × *ceil*(*log*2(*l*)) + 1 *with l* ∈ ℕ steps with l being the length of the path to be traversed until an obstructed line-of-sight is found. This new method allows the algorithm to quickly find the longest straight unobstructed lines between voxels in convoluted spaces and skip unnecessary voxels in open unobstructed spaces, making it the preferred method for this use case. A representation of the binary jump search is shown in Figure 5.

**Figure 6:**
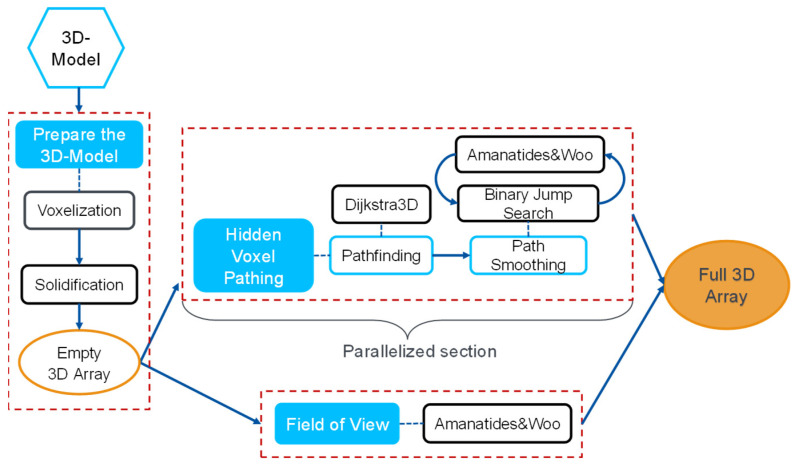
Process flowchart of the pathfinding algorithm.

Next, the Amanatides and Woo algorithm determines the shortest path between these voxels, resulting in a more accurate path between the starting point and destination. Additionally, the process of pathfinding is parallelized to increase the speed. The complete process flowchart is shown in Figure 6.

## 4. Results

### 4.1. Optimizations for Increased Speed

First, a line-of-sight test was performed for each of the four receiving sensors; namely, sensors 6, 13, 22, and 23. Table 1 displays the number of voxels within the line of sight of each sensor and the corresponding number of hidden voxels. The preprocessing step to find the field of view for each sensor not only increases the accuracy over Dijkstra’s algorithm, but Table 1 shows that it also saves significant time compared to running full pathfinding and optimization for the full array.

It is important to note that the full simulation was an approximation, an average time for several tests where only one slice of the array was traversed instead of the whole power transformer’s array. This allowed for randomized and targeted tests for the slices with the longest runtimes and those with the shortest.

It is also important to highlight that in this test, the pathfinding was performed with an unoptimized version of Dijkstra’s algorithm, which explored all nodes anew for every new target passed to it. This is not to be underestimated, given the number of nodes expanded each time. The unoptimized version of Dijkstra refers to the implementation of Dijkstra for the array without the postprocessing to the output path. This path was smoothed afterward to receive a much more accurate distance to the target. In addition, the field of view was not left unoptimized, meaning it did not utilize Amanatides and Woo’s algorithm for its completion but utilized a shorter and faster version of the line-of-sight algorithm to save time and show the potential for optimization. The line-of-sight algorithm utilized for the field-of-view creation was implemented fully in Python, without utilizing any other programming language and being written in a Pythonic manner.

One last important note on this test is that all algorithms were run on one thread of the previously mentioned processor, creating room for improvement via multithreading, which depends on the type and model of the processor used. In this case, allowing for preprocessing not only yielded the most accurate results possible for the nodes within the field of view but saved a significant amount of time compared to the full unoptimized implementation of Dijkstra’s algorithm. The impact of these optimizations is shown in Table 1.

### 4.2. Speed Comparison against Simulation and Experiment

Calculation speed is an important factor for data retrieval; with faster calculations, more data can be produced and processed. The speed comparison presented here can only be applied to the algorithm and the simulation, for the time to run an experiment is variable and dependent on several external factors. Thus, only the algorithm and the simulation can be compared, both running until completion on the same setup.

It is important to note that the simulation does not only calculate distances, as is the case with the algorithm, but distances are the only data being extracted from it for this use case. One run for the simulation consists of selecting one source and simulating a PD UHF wave, which will expand until it reaches the sensors. For this, the sensors must be modeled in 3D space and imported as part of the transformer. This way, the PD from one source can be traced towards as many sensors as modeled. This simulation takes, on average, 4.5 days to complete one run.

In addition, the algorithm traces a path from all free voxels towards one sensor. This process constitutes one run for the algorithm. The algorithm has to be run as many times as there are UHF sensors, i.e., four in this case. Each run for the algorithm takes, on average, 2.5 days, which means 10 days for all four sensors to compare their speed to the simulation directly.

Although the simulation seems to be faster, i.e., 4.5 days compared to 10 days of processing, the simulation only traces the path between one source and the four sensors. However, the algorithm traces the path from all available free voxels, where each voxel is a possible PD source, towards the four sensors, thus resulting in an overall speedup in comparison to the simulation.

### 4.3. Accuracy Comparison against Simulation and Experiment

Finally, the accuracy of the measurements obtained from all three methods can be compared. This section compares the results from utilizing four sensors as signal receivers and the rest as signal emitters. The total travel distance was measured for each sensor and each measuring method. Due to the high level of information available, the following results are presented in terms of total traveled distance in cm, as opposed to time differences of arrival, as is common in these kinds of measurements. The results can be converted into time of flight by multiplying the distance by the speed of light in the corresponding dielectric medium. Due to the homogeneity of the medium and, therefore, its properties throughout the traveled space, all signals travel at the same speed, making the traversed paths of equal cost throughout the transformer. Therefore, and because of the ease of visualization of the traveled distance and traversed path, the total traveled distances and their differences will be utilized to assert the accuracies of the given methods.

**Figure 7:**
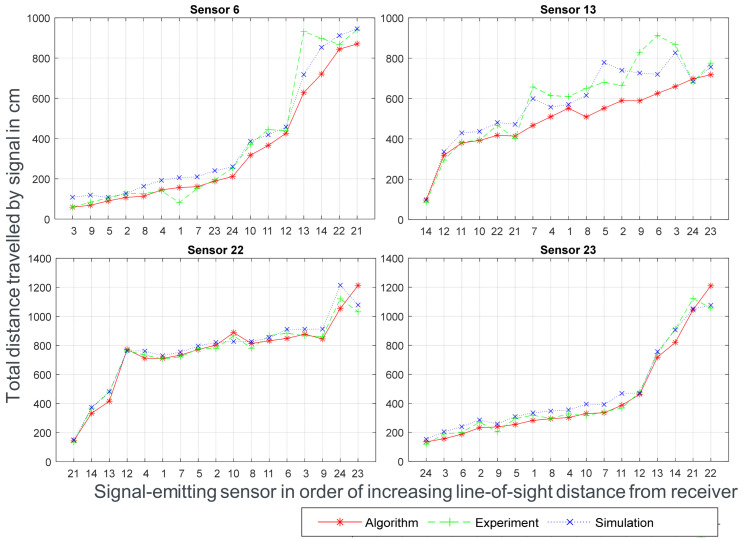
Comparison of travel distances for the pathing from the given sensors towards the respective receiving sensor for the algorithm, simulation, and experiment.

Figure 7 shows the results of these measurements for all given receiving sensors. As can be seen, discrepancies between all methods are present, but the trends of the signal propagation observed in the experimental data are well replicated in the algorithm and simulation. The electromagnetic simulation carried out in CST can over- or underestimate propagation distance when compared to the experiment. Meanwhile, the algorithm, most probably due to the low level of precision of the underlying voxelized model, namely voxels of 1 cm^3^, measured shorter distances overall than the simulation and experiment. All Euclidian distance measurements by the algorithm have travel distances shorter or equal to the ones measured by the simulation and the experiment, averaging −1.48 cm, proving that the algorithm possesses an accurate measuring capability and the voxelized environment is a good representation of the 3D model of the transformer in question. Three different types of signal propagation were considered to compare sections more precisely: a signal with a line-of-sight distance (direct propagation), a signal originating from the front of the transformer and traversing to the back or vice versa (indirect propagation), and finally, a signal traversing the whole transformer on its longest side (lateral propagation). As can be seen in Figure 7 and comparing with the utilized sensors, all signals received from sensors 1–9 and traveling towards sensor 6, signals originating from sensors 10–14 and traveling towards sensor 13, and the combinations 21–22 and 23–24 have a direct line of sight, and these are direct propagations. Signal combinations 22–23, 22–24, 21–23, and 21–24 are lateral propagations, spanning the largest distance between signal emitter and receiver. The rest of the signals are categorized as indirect propagations, which must follow a path through the winding blocks between the front and rear tank walls of the transformer.

As can be observed from receiving sensor 6, the first few points on the chart have a good correlation and low discrepancy among the algorithm, simulation, and experiment, which happens with direct propagation, as seen with combinations 23–24 and 21–22. Lateral propagation, in the case of combination 22–23, displays an inverse behavior, with the algorithm overestimating the travel distances instead of underestimating them the way it generally does. This behavior can easily be explained by the inaccuracy of the underlying path taken (in this case, pathing with a traditional pathfinding algorithm), which can measure the same distance when taking two different paths but with one of them having a longer true distance. This inaccuracy is more pronounced the longer the path is, thus leading to overestimations of distance. Similarly, the inaccuracies from indirect propagations can be explained by the increased complexity of the path taken, as shown in the chart for sensor 13 in Figure 7. Increased inaccuracy can be observed, especially for signals from sources 3 to 9, where the algorithm measures a much smaller travel distance than the other two methods.

**Figure 8:**
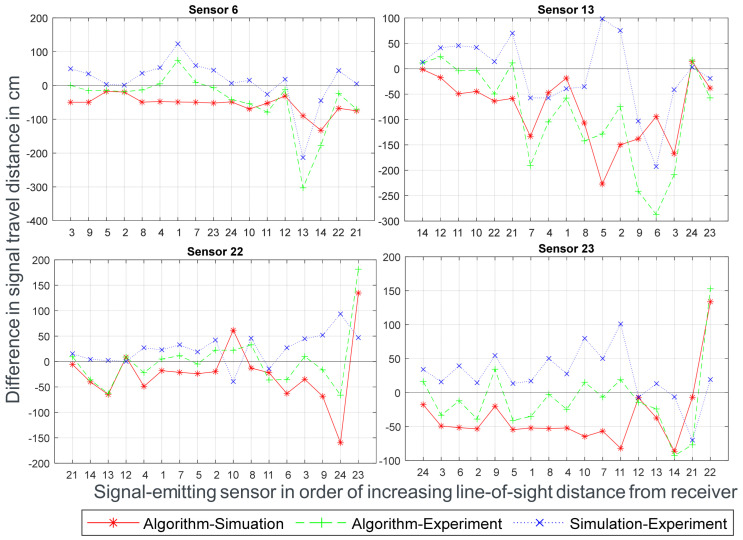
Comparison of travel distance differences for the pathing of the algorithm and simulation with respect to the experiment and each other. The y = 0 line signifies no difference in signal travel distance between each method.

Figure 8 compares the results of the algorithm and simulation to the results of the experiment and to each other by showing the difference in distance traveled by the signals from each source across the three methods. On comparing the algorithm with the simulation, as previously observed, the travel distances from the algorithm are almost always shorter than the ones from the simulation. On average, the distances traveled by the signals in the algorithm are 48.58 cm shorter. Additionally, the standard deviation of the distance difference is *stdev*_Algo-Sim_ = 55.81 cm.

The experiment is the closest data to the real-world scenario. However, measurement errors can still occur and the results should not be taken as absolute or perfect measurements. The lack of stability of the algorithm’s data can be seen in the extremes, where the algorithm always presents the largest absolute distance difference. When comparing the sum of the differences from all sensors, the standard deviation and the average deviation presented by the algorithm are 45% higher than the simulation; namely, *stdev*_Algo-Exp_ = 81.34 cm and *stdev*_Sim-Exp_ = 56.14 cm. However, the average of the algorithm’s data, at 21.28 cm, is much lower than the average of the simulation’s data, at 70.4 cm. This discrepancy can also be explained by the algorithm’s extremes, where correcting these leads to a lower standard deviation and average deviation than the simulation, which is precisely the case for sensors 22 and 23 when correcting for the extreme values in their data.

Next, the different PD sources were localized using the TDOA method with information obtained from the algorithm, experiment, and simulation, and the results are shown in Table 2. Additionally, the localization error was calculated using the distance formula, where the distance d between two points A (x_1_, y_1_, z_1_) and B (x_2_, y_2_, z_2_) is given by the following formula [29].


(1) $$d=\sqrt{{\left(x_{2}-x_{1}\right)}^{2}+{\left(y_{2}-y_{1}\right)}^{2}+{\left(z_{2}-z_{1}\right)}^{2}}$$

It can be observed that the algorithm has a lower average localization error of 107.81 cm compared to the other two methods, which have average localization errors of more than 180 cm. The reason for the difference in the average localization error could be attributed to the larger deviation observed in the algorithm when compared to the experiment and simulation in the case of sensor 13, as shown in Figure 7 and Figure 8. Another reason could be the fact that the signals consistently travel shorter distances in the algorithm than in the other two methods.

**Figure 9:**
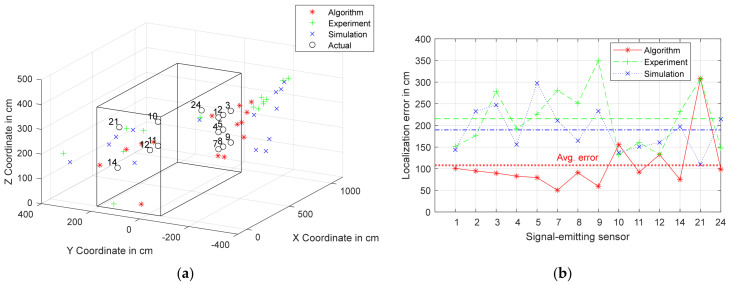
(**a**) Coordinates of PD sources calculated from the algorithm, experiment, and simulation against the actual coordinates; (**b**) localization error for each PD source obtained from each method along with the average error.

The algorithm was the least accurate in localizing PD source 21, with a localization error of 308.34 cm, whereas the experiment and simulation were least accurate in localizing PD sources 9 and 5, respectively, with localization errors of 349.27 cm and 297.57 cm, respectively. It is also apparent on analyzing each coordinate of the calculated locations that the x coordinates in all three methods are more accurate than the y and z coordinates. The calculated positions of the PD sources obtained from all three methods are plotted with the actual positions in Figure 9a, and the increased error along the y and z axes can be observed. Additionally, the localization errors of the PD sources obtained from each method are plotted in Figure 9b, along with the average localization error of each method. The lower error of the algorithm can be clearly observed.

## 5. Conclusions and Outlook

With the importance of power transformer PD monitoring and the rise of AI applications, it is necessary to quickly generate large amounts of data while being accurate. Although powerful and precise, current software cannot process the amount of data an AI needs for its training and validation sets. This paper introduces the idea of utilizing pathfinding algorithms to create TDOA data for use in training ANNs.

While it is true that the utilization of voxelized 3D environments in combination with traditional pathfinding algorithms introduces vast amounts of inaccuracies, when compared to real-world measurements, the simple introduction of Amanatides and Woo’s fast voxel-traversing algorithm helps to minimize these problems. Its utilization can not only be within the context of line-of-sight paths but can also smooth and adjust the path given by a traditional pathfinding algorithm.

The algorithm was tested on a voxelized 3D model of a 300 MVA transformer, and the results of the proposed algorithm were also compared with the results obtained from electromagnetic simulations and experiments. It was found that while the algorithm still over/underestimates certain paths, its accuracy is comparable to the accuracy of the simulation and, in some cases, even surpasses it. Comparisons to the data from the experiment and the simulation reveal that the algorithm in its current state can provide precise enough data in most situations. However, the spread of its data points must still be optimized. Overall, the algorithm has sufficient accuracy to reproduce the experimental results up to certain propagation distances, with the accuracy decreasing as the distance increases.

The localization error of the algorithm was compared with those of the experiment and simulation, and it was observed that the algorithm is more accurate than the other two methods. The reason for the lower localization error was the shorter distance traveled by the signal in the algorithm.

The algorithm’s accuracy was not achieved at the expense of speed. Certain optimizations and its modular design allow for even shorter runtimes. Being faster than the simulation while having comparable accuracy, this algorithm certainly has the potential to be useful for power transformer monitoring and may allow for AI tools to be created for this purpose. However, the deviation from the experiment and the simulation could be improved by using a mesh or a non-uniform grid instead of a voxelized environment. The use of a more accurate environment would, in turn, improve the accuracy of the path’s length by improving the algorithm’s pathing. As a result, new pathing possibilities emerge, such as the use of curves to define the path taken around an obstacle, like a cylinder, instead of stacking straight lines in a row. This demonstrates that improving one part of the system could open possibilities for further improvement that were not previously available or useful.

## Figures and Tables

**Table 1:** Impact of the time saved with the implementation of the field-of-view preprocessing for the entire power transformer.

Sensor	Voxels within the Field of View	Voxels within the Field of View as a Percentage of Total Voxels to Traverse	Number of Hidden Voxels	Time Taken for Field-of-View Calculation	Approximate Time Saved against Unoptimized Dijkstra’s Algorithm
Sensor 6	12,216,151	17.241%	58,639,051	4525.13 s (75.4 min)	2450 h
Sensor 13	8,904,782	12.56%	60,528,490	7688.4 s (128 min)	1786 h
Sensor 22	19,568,884	27.618%	51,286,318	2891.8 s (48.2 min)	3924 h
Sensor 23	11,704,256	16.518%	59,150,946	4157.8 s (69.3 min)	2347 h

**Table 2:** Localization errors of the algorithm, experiment, and simulation.

PD Source	Actual Coordinates	Localization from Algorithm		Localization from Experiment		Localization from Simulation	
		Coordinates	Error in cm	Coordinates	Error in cm	Coordinates	Error in cm
1	685, −48.75, 270	699.36, −129.83, 328.05	100.75	694.94, −187.16, 331.35	151.72	699.13, −188.51, 299.32	143.5
2	740, −48.75, 270	788.06, −129.08, 283.97	94.64	758.02, −198.32, 361.33	176.17	792.53, −245.48, 382.51	232.64
3	830, −48.75, 270	866.66, −120.18, 309.9	89.66	853.51, −274.78, 431.81	278.97	887.69, −246.7, 406.23	247.13
4	685, −48.75, 211	700.78, −119.7, 250.65	82.79	710.77, −202.28, 323.67	192.18	730.4, −185.76, 151.72	156.03
5	740, −48.75, 211	761.83, −116.6, 246.06	79.43	753.83, −220.39, 357.39	226.01	770.93, −274.1, 402.86	297.57 ^2^
7	685, −48.75, 142	718.55, −60.82, 106.35	50.42 ^1^	698.66, −227.69, 358.45	281.17	709.93, −247.35, 209.17	211.12
8	740, −48.75, 142	799.7, −7.26, 87.52	90.84	755.2, −207.35, 336.41	251.36	792.16, −204.99, 138.38	164.76
9	830, −48.75, 142	834.83, −100.5, 170.46	59.26	843.44, −256.96, 422.1	349.27 ^2^	885.59, −216.57, 294.19	233.28
10	718, 208.75, 207	816.6, 261.32, 98.74	155.58	795.59, 294.55, 143.37	132.03 ^1^	760.37, 325.05, 147.98	137.13
11	718, 208.75, 110	759.21, 290.12, 97.45	92.07	773.46, 355.12, 146.26	160.67	760.99, 318.97, 16.57	150.75
12	623, 208.75, 110	680.78, 325.18, 84.36	132.48	665.25, 330.38, 79.13	132.41	666.3, 361.68, 132.28	160.5
14	241, 208.75, 110	191.68, 264.12, 121.47	75.03	94.69, 378.74, 169.3	232	115.87, 359.31, 133.03	197.12
21	0.05, 118.5, 333	−38.46, 15.61, 44.89	308.34 ^2^	−103.68, 104.89, 44.34	307.03	−95.78, 127.39, 279.39	110.17 ^1^
24	949.95, 111.5, 225	1039.87, 100.32, 187.68	98	1075.33, 160.99, 164.68	147.67	1134.01, 183.15, 141.11	214.59
		Average error in cm	107.81		215.62		189.73

^1^ Minimum localization error; ^2^ Maximum localization error.

## Data Availability

The data presented in this study are available (subject to applicable restrictions) on request from the authors.
